# Structural insights into AVR-Rmg8 recognition mechanisms by the wheat blast resistance gene *Rmg8*

**DOI:** 10.1038/s41598-025-28559-5

**Published:** 2025-12-04

**Authors:** Soharth Hasnat, Tahsin Islam Sakif, M. Nazmul Hoque, Dipali Rani Gupta, Soichiro Asuke, Tofazzal Islam

**Affiliations:** 1 Institute of Biotechnology and Genetic Engineering (IBGE), Gazipur Agricultural University (GAU), 1706 Gazipur, Bangladesh; 2 Department of Gynecology, Obstetrics and Reproductive Health, Molecular Biology and Bioinformatics Laboratory (MBBL), GAU, 1706 Gazipur, Bangladesh; 3 Keck Graduate Institute, Claremont, CA 91711 USA; 4 Graduate School of Agricultural Science, Kobe University, Kobe, Japan

**Keywords:** MoT, *Rmg8*, AVR-Rmg8, PKC domain, MCTP kinase, Molecular mechanisms, Membrane dynamics, Computational biology and bioinformatics, Molecular biology, Plant sciences

## Abstract

Wheat blast disease, caused by the *Triticum* pathotype of *Magnaporthe oryzae* (MoT), poses a significant threat to global food security. The blast resistance gene *Rmg8*, recently isolated from a hexaploid wheat cultivar, strongly confers resistance to all Bangladeshi and Zambian MoT isolates that carry the eI type of AVR-Rmg8. However, the molecular interactions underlying this recognition at the protein level remain poorly understood. In this study, we elucidated the structural and biological characteristics of RMG8 proteins and their recognition of the AVR-Rmg8 effector proteins using computational biology approaches. Amino acid sequence comparison of four AVR-Rmg8 types revealed that only three amino acid residues distinguish the eI type of AVR-Rmg8, which induces a higher level of resistance conferred by RMG8. The most intriguing finding of this study is that only the eI type effector interacts with ATP through the Pro26 residue, a feature not present in the other AVR-Rmg8 types. We identified that the Protein Kinase C (PKC) domain of RMG8, where proline dependency mediates the phosphorylation of a serine residue, is involved in the strong recognition of the eI type of AVR-Rmg8. Phylogenetic analyses indicated that RMG8 might have evolved from proteins closely associated with plant signaling pathways. Although *Rmg8* is an atypical resistance gene, our data suggest that it may function as a hub in the plant defense network, as it is a type of nuclear membrane protein, specifically a calcium-dependent multiple C2 domain protein with transmembrane regions (MCTP) kinase, which integrates signaling for effector recognition. Taken together, our study provides detailed insights into the molecular recognition mechanism between AVR-Rmg8 and RMG8, which is expected to aid in wheat blast resistance breeding. Future studies involving the purification and structural characterization of MoT effector proteins and *Rmg8* gene products are necessary to validate these findings.

**Supplementary Information:**

The online version contains supplementary material available at 10.1038/s41598-025-28559-5.

## Introduction

Wheat is a staple crop grown on approximately 220.7 million hectares worldwide, with a total production of 799 million tons. However, wheat production faces significant challenges due to various diseases, including wheat blast caused by *Magnaporthe oryzae* pathotype *Triticum* (MoT) (Synonym: *Pyricularia oryzae*)^1^. This disease has emerged as a substantial threat to wheat production, particularly in warm and humid region. Wheat blast is now a major threat to global food security. This disease first emerged in Brazil in 1985 through a host jump of the *Lolium* pathotype of *M. oryzae* (MoL) or its relatives^2,3^. This devastating disease was recently introduced into Bangladesh in South Asia^4^ and Zambia in Africa^5^ likely through grain trading. During pathogen infection, plants activate defense mechanisms, such as the hypersensitive response (HR), driven by the recognition of pathogen effectors by plant-encoded resistance (R) proteins. Therefore, identifying and understanding R-genes are crucial for developing effective disease management strategies^2^. R-genes, often encode nucleotide-binding leucine-rich repeat receptors (NLRs), detect specific pathogen effectors that otherwise suppress plant immune responses^3^.

To broaden the spectrum of detected pathogens, NLRs have diversified through gene duplication and variations in leucine-rich repeats (LRRs), enabling recognition of new effectors and forming complex signaling networks^6^. *Rmg8*, a gene for resistance to wheat blast found in a hexaploid wheat cultivar S-615, has been cloned as a first cloning case of wheat blast resistance gene^7^_’_^8^. Interestingly, *Rmg8 *gene consists of two spliced variants, both encoding a putative chimeric protein that includes a serine/threonine kinase with multiple C2 domains and transmembrane regions (kinase-MCTP). Both spliced variants (RMG8-V1 and RMG8-V2) are necessary for resistance, as it has been demonstrated that the absence of one variant cannot confer resistance against MoT^7^._’_^8^. Cloning of *Rmg8* also revealed that *Rmg7*, a gene for resistance to wheat blast found in tetraploid wheat cultivar St24, is a homologous gene of *Rmg8* and also an allele of *Pm4*, a gene for resistance to wheat powdery mildew^7,9^. Phylogenetic studies suggested that *Rmg8* and its variants found in tetraploid and hexaploid wheat populations have emerged to expand their range of target pathogens^7^. AVR-Rmg8 in MoT isolates is roughly classified into two types, the eI and eII type of AVR-Rmg8 based on amino acid sequence. AVR-Rmg8 in MoL is roughly classified into two types the eL1 and eL2 type of AVR-Rmg8. In MoT, the eI type of AVR-Rmg8 is strongly recognized by *Rmg8*, but the eII type of AVR-Rmg8 partially evade recognition^10^_’_^11^. Noteworthy, all MoT isolates collected in Bangladesh and Zambia carry the eI type of AVR-Rmg8 suggesting that *Rmg8* may be beneficial in Bangladesh and Zambia^10^. Notably, *Rmg8* is distinguished by its effectiveness against multiple MoT isolates across different developmental stages and environmental conditions including higher temperature, highlighting its potential utility in diverse agricultural settings especially in Bangladesh and Zambia^12^. Despite advancements, the molecular interactions and structural details underlying *Rmg8*’s specific recognition of pathogen effectors remain poorly understood, as does the signaling pathway through which *Rmg8* imparts resistance^7,9^.

This study aimed to employ advanced computational biology to investigate the interaction between RMG8 and AVR-Rmg8. Nowadays, computational biology is being routinely used to reveal insights into biological mechanisms^13^. Computational platforms like those offered by Schrödinger LLC have proven to be a cost-effective and efficient way to design and develop biological environments, providing significant insights within a limited context timeframe^14^. Our results demonstrate that the eI type of AVR-Rmg8 binds with high affinity to the PKC domain of RMG8, facilitated by ATP binding to Pro26 residue of RMG8. Furthermore, RMG8, a calcium-dependent MCTP kinase, specifically targets the eI type of AVR-Rmg8, with three amino acid differences critical for this recognition. Overall, our results for provide significant insights into the molecular mechanisms underlying the interactions between AVR-Rmg8 and RMG8.

## Results

### RMG8 and AVR-Rmg8 interactions mediated by three mutated amino acids

**Fig. 1:**
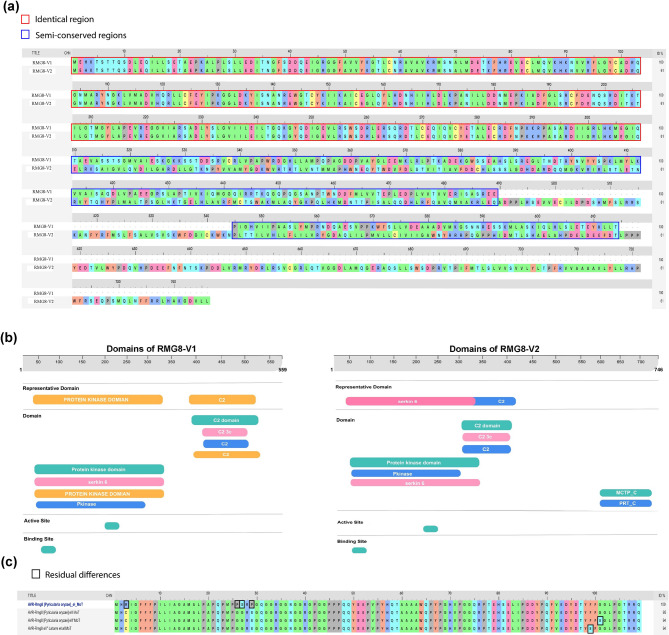
Conserveness of RMG8 among splicing variants (RMG8-V1 and RMG8-V2) and AVR-Rmg8 types distributed in MoT. (**a**) Variation and conserveness in RMG8-V1 and RMG8-V2. (**b**) Annotated biological functions with representative domains in both variants of RMG8. (**c**) Differences and conservation among different types of AVR-Rmg8 proteins.

To understand the similarities between the two variants (variant 1, denoted as RMG8-V1, and variant 2, denoted as RMG8-V2) of RMG8, we performed a multiple sequence alignment (MSA). RMG8-V1 contains 559 amino acids (BET03927.1), and RMG8-V2 contains 746 amino acids (BET03928.1). Using the MSA module of BioLuminate revealed that both variants have 68% conserved regions. Specifically, both variants have 309 identical residues positioned at the beginning of the sequence, containing a protein kinase C (PKC) domain (Fig. 1a). The rest of the sequence of RMG8-V1 falls in the semi-conserved region, and unmarked regions fall in gap regions identified by the MSA viewer (Fig. 1a). Additionally, a membrane-targeting C2 domain that is rich in aspartate and dependent on Ca^2+^ was identified in the semi-conserved regions of both variants, exhibiting 60% identity. Notably, an extra domain associated with the PRT_C (Plant phosphoribosyl transferase) superfamily was found exclusively at the C-terminus of RMG8-V2 (Fig. 1a and b). Both RMG8 variants possess identical active and binding site residues within their PKC domains (Fig. 1b). Overall, the conservation and domain analysis provide a comprehensive understanding of the biological and molecular roles of RMG8-V1 and RMG8-V2.

On the other hand, the conserveness analysis of AVR-Rmg8 helped to reveal the variation among the different types of AVR-Rmg8. All the AVR-Rmg8 effector types have 109 amino acid residues that are around 95–96% conserved. The eI type of AVR-Rmg8 possesses only 4 differences at Arg3, Pro26, Ser27, and Pro29 (Fig. 1c).

### Evolutionary analysis reveals higher leucine content in RMG8

**Fig. 2:**
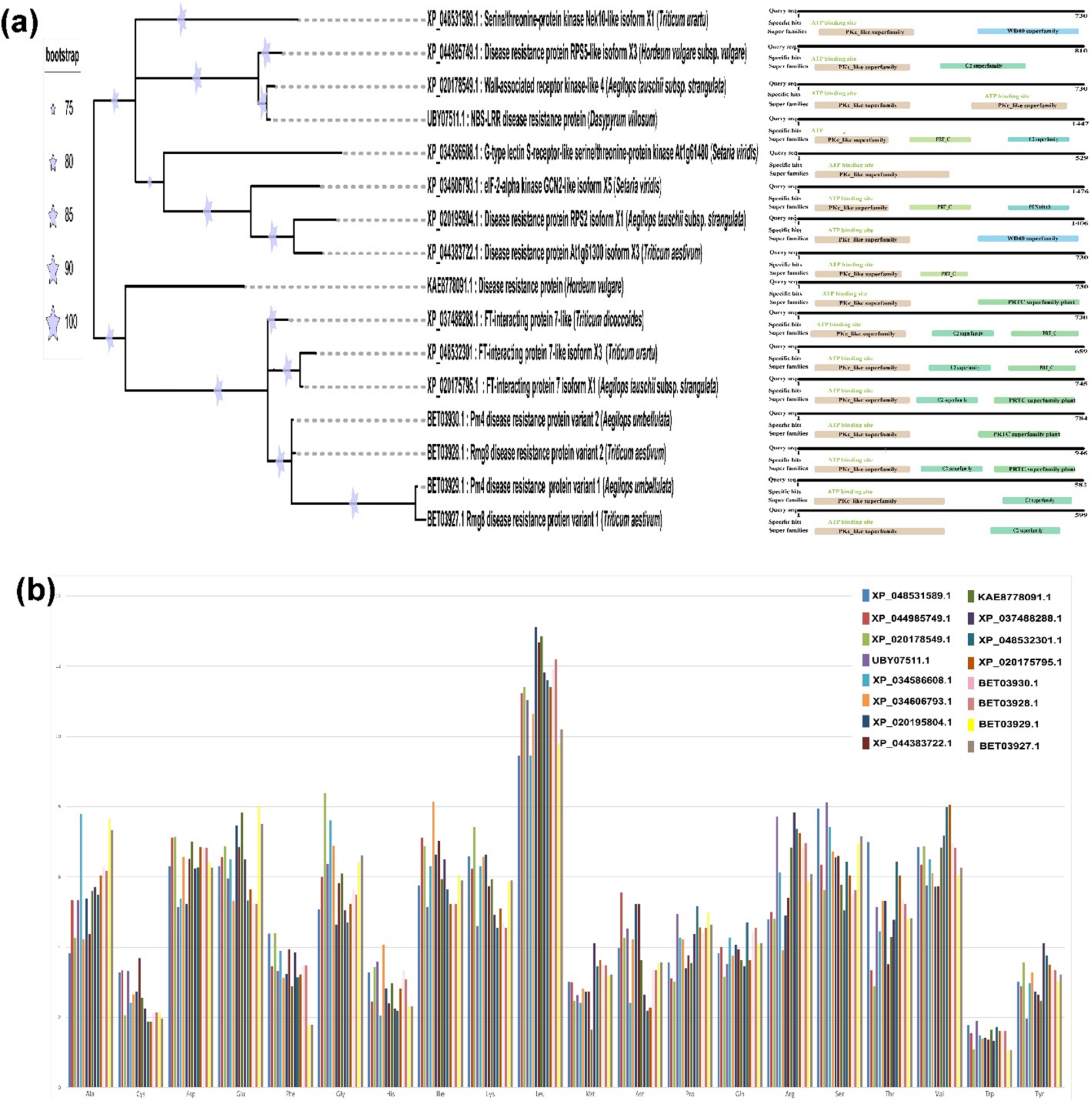
Evolutionary ancestor of RMG8 protein. (**a**) Both variants of RMG8 demonstrate strong ancestral relations with the PKC domain-containing proteins, which have a strong background in the signaling pathway. (**b**) Amino acid ratio of 16 evolutionarily related proteins demonstrates the higher leucine residues in the PKC domain of both variants of RMG8.

To find the ancestral relation of RMG8 with the other proteins, we have taken a total of 500 proteins from the output of BLASTp that share sequence similarities with both variants of RMG8. Based on retrieved domain information, proteins with higher conservation and having at least a PKC domain were chosen for phylogenetic analysis. RAxML (Randomized Axelerated Maximum Likelihood) analysis with 1000 times bootstrapping demonstrated a deep ancestral relation between RMG8 and other proteins (Fig. 2a). Variants of the disease-resistant protein PM4 of *Aegilops umbellulata* showed the highest conservation with RMG8 variants. After the PM4 proteins, a close relation was found with the FLOWERING LOCUS T (FT)-interacting protein (Fig. 2a). The rest of the eight proteins of the phylogenetic tree were detected as ancestral to RMG8. The phylogenetic tree was identified as ancestral to RMG8, each supported by a bootstrap value of 75, indicating that their evolutionary clustering is reliable and suggests a conserved evolutionary relationship (Fig. 2a). The amino acid residue frequencies of RMG8 were compared with other ancestral proteins (Fig. 2b). Interestingly, leucine content was higher than any other amino acid as an individual amino acid in all chosen proteins as well as leucine repeats was predominant in PKC domain (Fig. 2b).

### Pathway analysis reveals RMG8 as a hub for regulating the defense signaling

There was no exact pathway determined for the RMG8 yet. However, a probable pathway for RMG8 was inferred from the KEGG database, which is related to the disease-resistant kinase protein of *Aegilops tauschii*, encoded by RMG8. The result showed that RMG8 follows a similar pathway to a leucine-rich disease-resistance plant protein (Fig. S1). The proposed tentative pathway provided a preliminary framework for understanding RMG8’s potential role, which described that after the secretion of the avirulent protein from the host, effector-triggered immunity is activated by the diverse types of disease-resistant proteins (Fig. S1). Subsequently, protein-protein interaction analysis in the STRING database suggested that RMG8 interacts with various types of proteins, and all of them are involved in cell signaling, stress regulation, and defense mechanisms (Fig. S2). Both the KEGG and STRING analysis described RMG8 as acting as a hub for regulating the defense signaling (Figs. S1-S2). The most frequent interactions of RMG8 were observed with serine/threonine phosphatase, AMPK activators, PP2Cs, and Pseudokinase. Interestingly, these four proteins have a strong association with other proteins identified through STRING analysis presented in Fig. S2.

### Structural analysis reveals divergence in RMG8 and AVR-Rmg8 proteins architecture

To understand the molecular architecture of RMG8 variants, 500 structures were generated for each variant using Rosetta. Protein structures with the lowest RMSD values for RMG8-V1 and RMG8-V2 were considered the best models. The top 50 structures of each RMG8 variant with the lowest RMSD values are available in Files S1-S2. For RMG8-V1, the RMSD values ranged from 0.87 to 11.34 (Table S1). In contrast, for RMG8-V2, the RMSD values ranged from 0.58 to 18.47 (Table S2). For further analysis, we selected the structure with the lowest RMSD value for both RMG8-V1 and RMG8-V2. Additionally, the calculated values for non-bonded interactions between different atom types, and the plots showing the error function versus the position of a 9-residue sliding window for both variants, were over 96%. The Ramachandran plot indicated that over 93% of residues fall within the most favored regions, which is ideally acceptable. Similar methods were applied to the four types of AVR-Rmg8, and the best structures were chosen based on RMSD values.

### RMG8 variants possess identical PKC domain with distinct functional and signaling features

**Fig. 3:**
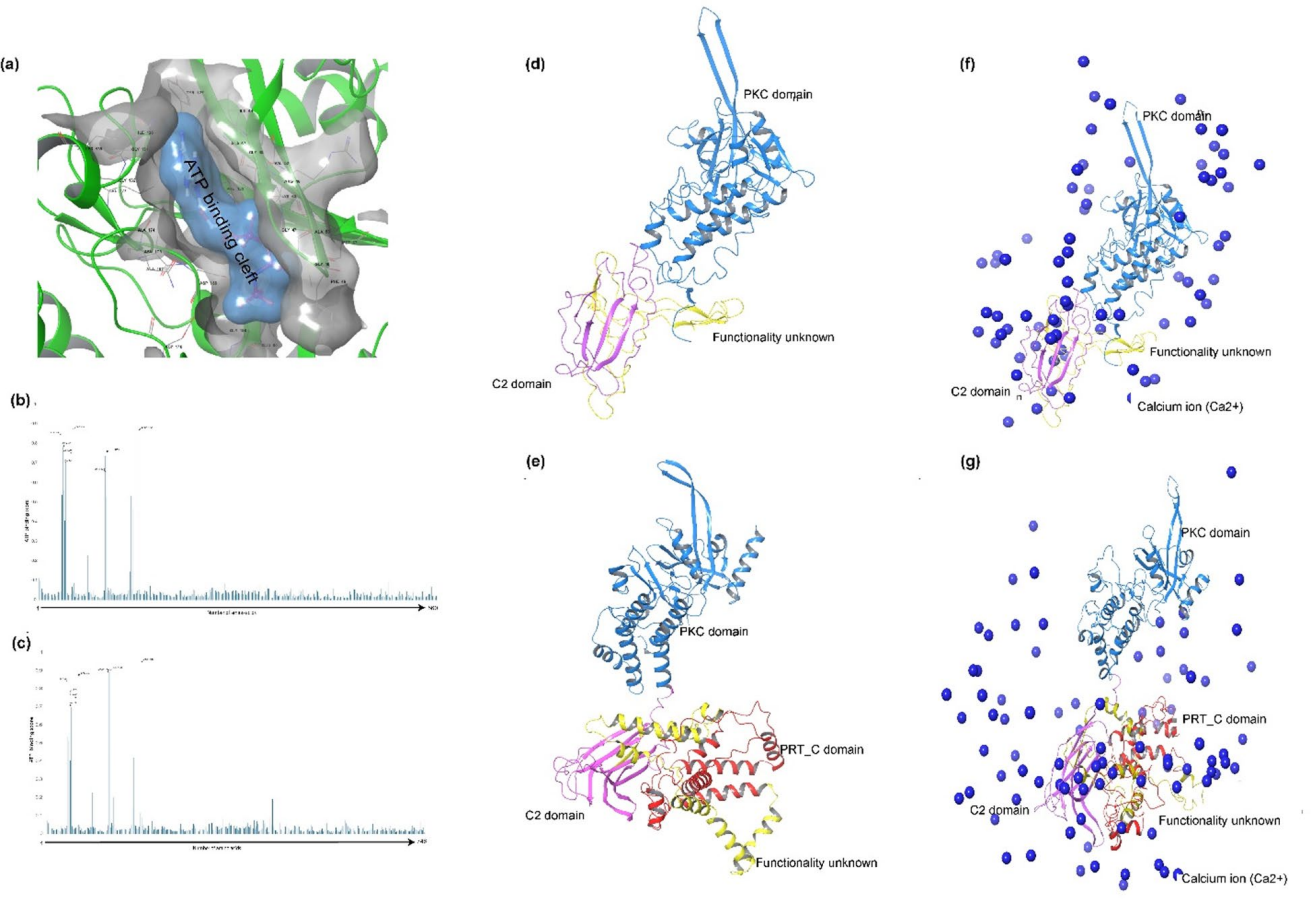
Molecular feature of RMG8 protein variants. (**a**) Location of ATP binding cleft in the PKC domain. (**b**) Probability score for the amino acid residues of PKC domain of RMG8-V1 involved in ATP binding. (**c**) Probability score for the amino acid residues of PKC domain of RMG8-V2 involved in ATP binding. (**d**) RMG8-V1 has two different domains with a functionally unknown region. (**e**) RMG8-V2 has an extra PRT_C domain than RMG8-V1 with a functionally unknown region. (**f**) Calcium up-taking capabilities of C2 domain of RMG8-V1. (**g**) Calcium dependency in RMG8-V2.

To understand the signaling pathway and recognition mechanisms of RMG8, we sought to uncover the molecular function of each domain within both RMG8-V1 and RMG8-V2. We found that the PKC domains in both variants are 100% identical (Fig. 1). In our in-silico molecular function analysis, we confirmed the kinase activity of the identical PKC domain by examining the ATP binding cleft, which exhibited higher affinities for ATP (Fig. 3a). The initial screening for the affinity of the PKC domain for ATP showed that the surface of the ATP binding cleft was covered by various amino acids (Fig. 3a). To know the involvement of particular amino acid residues in the ATP binding process, we performed E2EATP analysis, and its assessments showed that amino acids Ile44, Gly47, Val52, Lys65, Cys24, Ile128, and Asp188 from both variants have more than 70% chances to interact with ATP molecule (Fig. 3b and c). Earlier, it was revealed that both variants of RMG8 carried Ca^2+^ dependent membrane-targeting modules C2 domains (Fig. 3d and e) and the characteristics of C2 domains were examined through the Metal Ion-Binding Site (MIB) predictor. The result of the MIB predictor demonstrated Ca^2+^ deposition at the C2 domain (Fig. 3f and g). RMG8-V2 has an extra PRT_C domain, which also showed affinities for Ca^2+^ (Fig. 3g). Another crucial molecular feature of RMG8 variants was that both of them were identified as nuclear membrane proteins through the Plant-mPLoc and TMHMM – 2.0 analysis. To investigate the signaling characteristics of AVR-Rmg8, we analyzed its signaling patterns and the presence of the signal peptide. Our findings, illustrated in Fig. S3, indicate that there were no significant differences in the signal peptide associated with AVR-Rmg8, nor did we observe variations in the strength of the signal peptide. This suggests that AVR-Rmg8 maintains consistent signaling behavior under the conditions tested, which may have implications for its functional role in the signaling pathways involved.

### Molecular dynamic simulation reveals effective recognition and kinase phosphorylation in RMG8

**Fig. 4:**
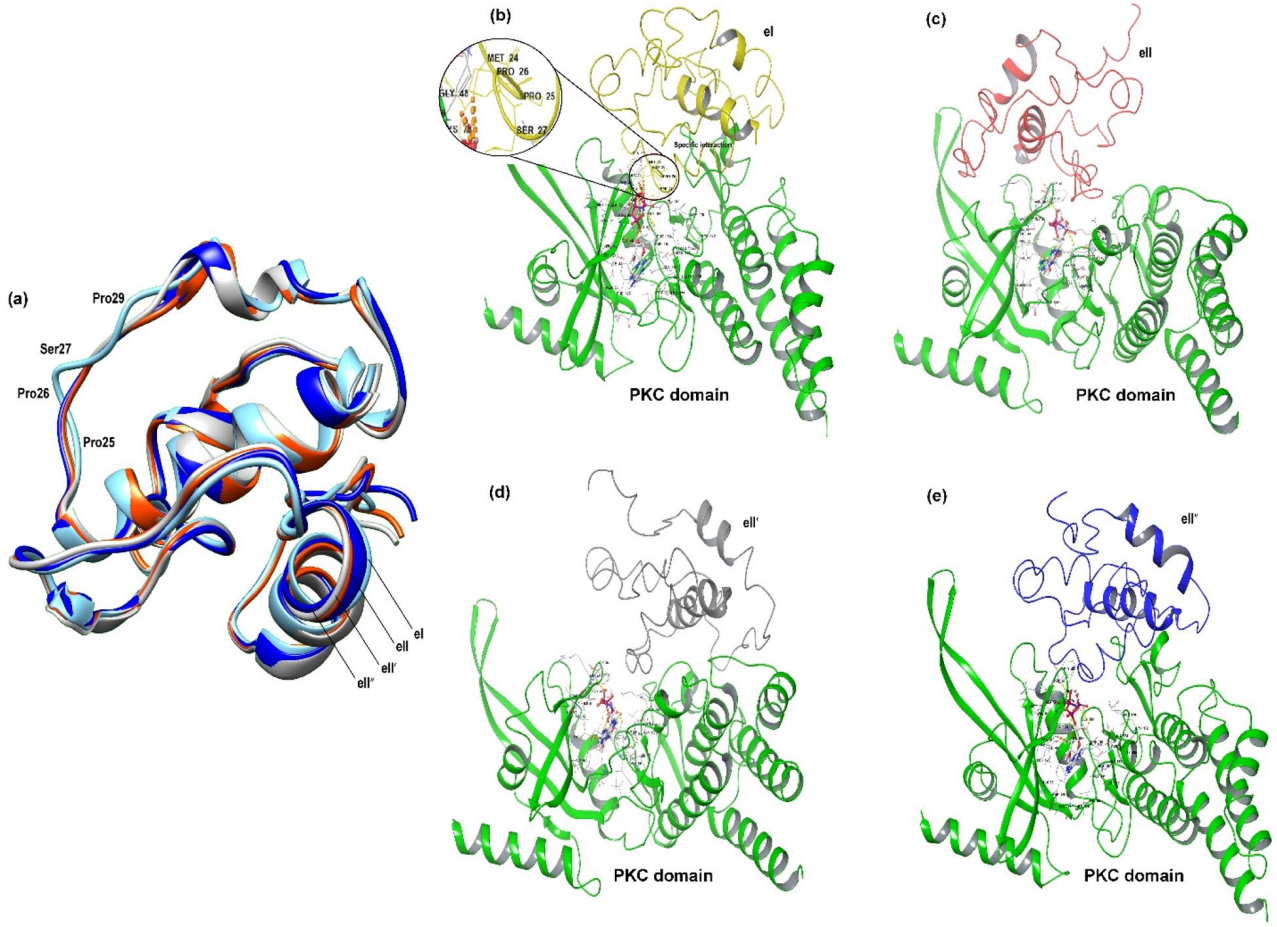
Effective recognition of AVR-Rmg8 by kinase phosphorylation. (**a**) Structural comparisons of 4 eI variants of Avr-Rmg8. (**b**) Specific recognition was found due to the proline-dependent phosphorylation mediated by the PKC domain of both variants of RMG8 protein. (**c**) No PKC domain-mediated phosphorylation was observed in the presence of ATP to eII. (**d**) No strong interaction was found in the presence of ATP with eL1 type. (**e**) Similarly, eL2 type cannot be phosphorylated by PKC domain.

In the MSA analysis, it was disclosed that the eI type has three residual differences with the other three types of AVR-Rmg8 (Fig. 1b). To determine the effect of these differences at the molecular level, structural comparisons were rigorously performed. The output of the comparison analysis revealed that the structure of the eI type of AVR-Rmg8 has four major changes at the residual position of Pro25, Pro26, Ser27, and Pro29 (Fig. 4a). To learn more about the effect of these discrepancies, we performed multiple screenings for each RMG8 and AVR-Rmg8 complex in the presence of ATP. Molecular dynamics simulations revealed that the ATP molecule maintained stable binding with the PCK domain in each complex, with Root Mean Square Deviation (RMSD) values remaining below 4 Å (Fig. S4). The Root Mean Square Fluctuation (RMSF) analysis indicated a maximum fluctuation of ~ 3.5 Å, consistent with stable protein flexibility. Likewise, the radius of gyration (rGyr) remained stable at approximately 4.5 Å, and the interaction free energy of the complexes reflected a robust interaction, indicating sustained structural compactness and extended stability of the complexes. A total of ten MM/GBSA free energy parameters were evaluated, and the negative ΔG_bind values confirmed the energetically favorable interactions (Fig. S4). The initial screening showed Arg46, Lys 65, and Asp 188 residues of the PKC domain interact with the ATP molecule (Figs. 4b-4e). The most intriguing finding of this study is that only the eI type effector interacts with ATP through the Pro26 residue, a feature not present in the other AVR-Rmg8 types. This distinct interaction likely enhances the strong recognition of the eI type by the RMG8 receptor, attributed to its unique Pro26 residue, which facilitates effective recognition and subsequent phosphorylation (Fig. 4b). However, a strong interaction between the eII, eII’, and eII’’ types and the PKC domain was not detected, although the results indicated that initial recognition may take place. The output of molecular dynamic simulation further confirmed the Kinase activity of the PKC domain and its phosphorylating capacity to eI type of AVR-Rmg8 (File S3). During the dynamic study, it was also observed that the Pro26 residue of eI type strongly interacts with the ATP molecule (Fig. 4b, File S3).

### RMG8 variants and membrane insertion reveal suitable and energetically favorable interactions

**Fig. 5:**
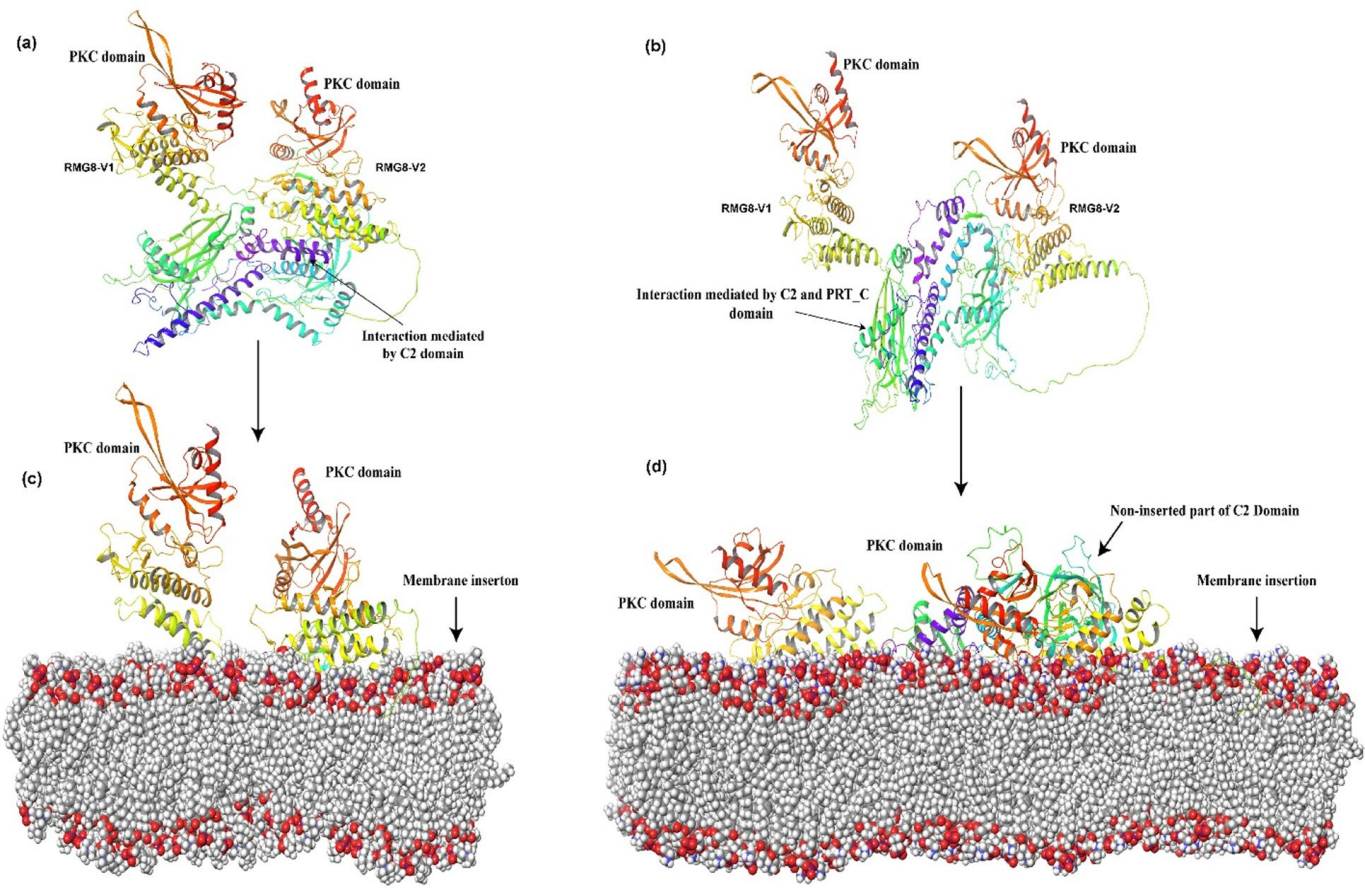
Dimerization and membrane insertion of RMG8 variants. (**a**) Interaction mediated by C2 domains of both variants. (**b**) Interaction mediated by C2 domain of RMG8-V1 and PRT_C domain of RMG8-V2. (**c**) Demonstration of membrane integration of RMG8 complex only mediated by C2 domains. (**d**) Membrane integration of C2 and PRT_C mediated RMG8 complex in the lipid bilayer system.

To understand why both variants of RMG8 are required for dimerization to confer resistance against MoT, we have screened their dimeric interaction using multifold protein-protein docking. We have found an outstanding interaction between both variants of RMG8 through the random screening process in BioLuminate. Performing the screening process, rotating 70,000 times returned 30 RMG8 complexes. After analyzing them, only two complexes were shown as convenient, considering their interaction strength and feasibility. We observed the involvement of different domains in forming complexes with RMG8 variants. One complex was formed through the interaction of C2 domains of both RMG8 variants, and the other was formed through the interaction of PRT_C and C2 domains (Fig. 5a and b). We conducted additional screenings without the C2 domain in RMG8-V1 and found no interactions between the two variants. In the absence of the PRT_C domain in RMG8-V2, a complex dimer was formed through the C2 domains of both variants. Thus, the most significant likelihood was that RMG8-V1 formed a complex with RMG8-V2 variant through two possible mechanisms before translocating into a lipid bilayer (Fig. 5c and d). C2 domain-mediated complex was inserted more into the lipid membrane compared to the PRT_C and C2 mediated complex. However, throughout the molecular dynamic trajectories, PRT_C and C2 were demonstrated as a suitable and energetically favorable RMG8 complex in the lipid bilayer system compared to the only C2-mediated complex (Fig. 5c and d, Files S4-S5).

### RMG8 membrane dynamics with eI type of AVR-Rmg8 reveals domain localization and kinase activity

**Fig. 6:**
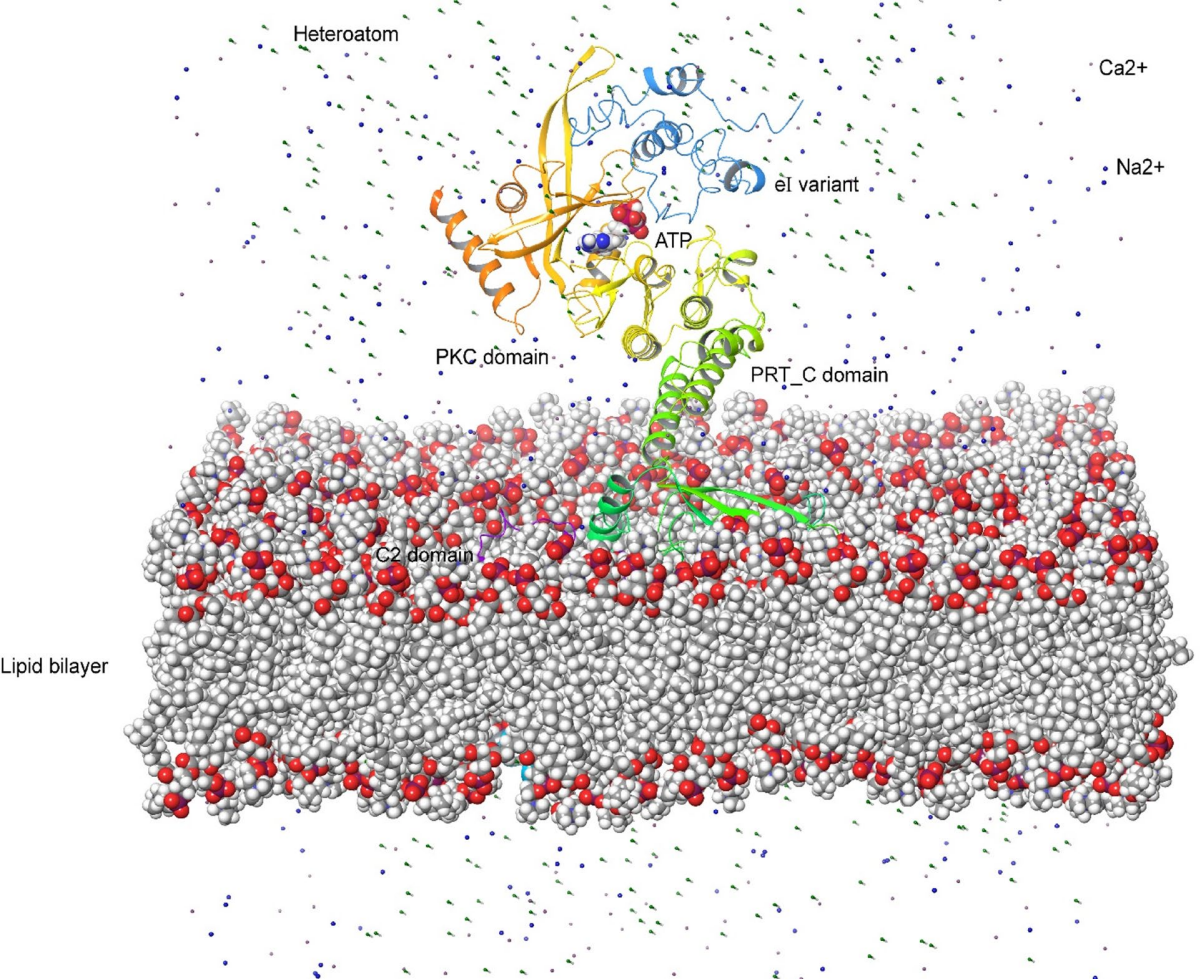
Membrane dynamics of RMG8-V2 while interacting with AVR-Rmg8. A live membrane model was constructed in the presence of effector AVR-Rmg8, Ca^2+^ Na^+^ and heteroatoms to test the biological activity of the RMG8 in the membrane system.

To capture the RMG8 interaction with the eI type of AVR-Rmg8 in the membrane system, a complete membrane was built up following the composition of an outer nuclear membrane. To avoid the limitation of capturing multiple interactions at a time, the complex was formed by a single variant, ATP and the eI type, and input into CHARMM-GUI. The membrane system demonstrated that the whole PKC domain fell on the outer side of the nuclear membrane, and the C2 domain and PRT_C domain fell inside the membrane regions (Fig. 6). The membrane system was only developed for RMG8-V2 variant, while RMG8-V1 has common domains except the PRT_C domain. To capture the dynamics of RMG8 proteins in the membrane system, we computationally created a living environment by adding ions like Na^2+^, Ca^2+^, and heteroatoms. In the membrane system, the PKC domain of RMG8-V2 exhibited kinase activity towards the eI types, indicating that the kinase domain of RMG8 may serve as a receptor for AVR-Rmg8 (Fig. 6, File S6). The activity of the PKC domain was captured in the membrane system with the interaction of the variant through the ATP molecule (File S6). A large portion of the other two domains fall inside the membrane system, and it was difficult to capture activity inside the membrane system. However, in the previous analysis, the C2 and PRT_C domains demonstrated affinities for Ca^2+^ ions. In the membrane system, we have found a similar demonstration (File S6). To confirm the demonstration of RMG8 in the lipid bilayer, we rigorously perform the dynamics of RMG8 in the membrane system. The output demonstrated the animation of RMG8 in the membrane system (File S7).

## Discussion

In this study, we elucidated the molecular mechanisms underlying the specificity of *Rmg8* gene products in recognizing AVR-Rmg8 effector proteins from MoT isolates found in Bangladesh and Zambia. Our computational analyses revealed three critical residue changes in the eI type of AVR-Rmg8 that are pivotal for effective recognition by RMG8. Among these, the substitution of glycine with proline at position 26 enables phosphorylation of adjacent serine residues an essential modification facilitating recognition. This feature, unique to the eI type, underlies the heightened specificity of RMG8 and was identified as a hallmark of strong resistance. Phosphorylation, a key regulatory mechanism in plant immunity^15^, thus plays a central role in RMG8-mediated blast resistance, as other AVR-Rmg8 types lacking Pro26 do not trigger the same response. We further demonstrated that RMG8 likely functions as a membrane-associated kinase, specifically a calcium-dependent multiple C2 domain protein with transmembrane regions (MCTP) that interacts with eI-type AVR-Rmg8 effectors. Although RMG8 is an atypical resistance gene, our structural and evolutionary analyses highlight its role as a hub in the plant immune network. Our phylogenetic study revealed that RMG8 shares catalytic motifs with serine/threonine-specific kinases, and its closest evolutionary relatives are signaling proteins in Triticeae plants (Fig. 2a)^12,16–21^. These proteins, like classical NLRs, are enriched in leucine residues, which are vital for effector recognition and defense activation^10,22,23^. We also identified ATP-binding clefts in the PKC domain of RMG8, further supporting its kinase function and suggesting its role in ATP-mediated signal transduction.

Another significant contribution of this study is revealing the interaction dynamics between RMG8-V1 and RMG8-V2 protein variants within the membrane. Using structural modeling and dynamic simulations, we demonstrated that resistance depends on a stable complex formed through the interaction of C2 and PRT_C domains within the membrane system. The absence of the C2 domain in RMG8-V1 prevented complex formation, confirming that both variants are essential for effective resistance. This mechanistic insight supports prior hypotheses^7,8,21,22^ and clarifies that while the C2 domain can mediate some interaction, the most stable and functionally relevant complex requires both C2 and PRT_C domains. RMG8-V2 uniquely carries the PRT_C domain, reinforcing its indispensable role.

At the molecular level, we found that RMG8 operates as a proline-directed serine/threonine kinase, capable of phosphorylating serine residues immediately adjacent to proline^24^. The presence of the PKC domain, with its high leucine content^25^, reinforces its classification among disease-resistance proteins, similar to other NLRs like Pm2h, Pm3a, and Pm4bh^23^. RMG8 functions within the nuclear membrane system, as well as the presence of the C2 and PRT_C domains, suggesting regulation by Ca²⁺/calmodulin complexes. These observations indicate that Ca²⁺ binding may be important for RMG8’s potential interactions with other immune proteins and its role in pathogen recognition. Additionally, predicted ATP-binding sites could support structural stabilization or conformational changes associated with the activation of RMG8. Importantly, our simulation studies are the first report of the RMG8 functions within the nuclear membrane system and are regulated by Ca²⁺/calmodulin complexes via its C2 and PRT_C domains. These novel findings not only clarify how RMG8 recognizes the eI type but also indicate how this interaction could lead to downstream activation of immune responses. Although we modeled these interactions using high-confidence structural prediction tools, it remains essential to validate these findings through biochemical and crystallographic studies. Nevertheless, the proline-dependent phosphorylation mechanism we uncovered introduces a new regulatory layer in RMG8 activation, enriching our understanding of plant immune signaling^26–28^. Our study positions RMG8 as a critical proline-directed kinase involved in conformational signaling cascades^24^. From a molecular breeding perspective, this mechanism opens new avenues for engineering more robust resistance traits in wheat. For example, breeders could target phosphorylation-prone motifs in the PKC domain to enhance RMG8 function. Screening wheat germplasm for favorable RMG8 variants or editing phosphorylation sites via CRISPR-Cas genome editing could improve wheat’s defense against MoT. Notably, while RMG7/Pm4 shares identical protein sequences with RMG8, its expression in different genetic backgrounds shows variation in resistance strength^2,29^. This suggests that RMG8’s effectiveness is influenced not only by its protein structure but also by host genomic context and epigenetic factors. A major limitation of our work is the absence of crystallized structures for RMG8 and AVR-Rmg8, which are needed to definitively confirm the interaction mechanisms we propose. Still, our findings provide compelling evidence that both RMG8 variants are necessary for MoT resistance and clarify the molecular basis of their cooperation a long-standing question following the cloning of the *Rmg8* gene^7, 8^.

## Conclusion

In summary, RMG8 stands as a promising defensive weapon against the MoT variant responsible for wheat blast in Bangladesh and Zambia. While its potential has been investigated at the genomic and transcriptomic levels, the precise biological functions of the RMG8 protein within molecular mechanisms remain largely unexplored. This study for the first time elucidated the possible biological mechanisms, using computational biology approaches, by which the RMG8 protein can recognize the eI type of AVR-Rmg8 and expanded our understanding of its specific effectiveness against this type of AVR-Rmg8. Our phylogenetic analysis highlights a strong ancestral connection between RMG8 and other disease-resistant proteins. The molecular architecture and membrane dynamics studies presented here underscore how *Rmg8* mediates robust defense mechanisms against MoT. This study demonstrates that the structural features and molecular functions of RMG8 are intricately linked to its ability to recognize and respond to pathogen effectors, thereby activating plant defense mechanisms. The findings of this study provide a crucial foundation for the future engineering of an enhanced RMG8 protein capable of offering broad-spectrum resistance in wheat against all MoT variants. Additionally, our work opens the door for further proteomic studies related to *Rmg8*. These advancements could significantly bolster wheat resistance, reducing the impact of wheat blast in affected regions like Bangladesh and Zambia and potentially preventing future outbreaks globally. Field trials to assess the efficacy of the *Rmg8* gene against wheat blast fungus in Bangladesh and Zambia are urgently needed to validate these findings and facilitate the deployment of this resistance strategy in agricultural practices. However, future research is needed to validate our findings by purifying and structurally characterizing MoT effector proteins and *Rmg8* gene products for precisely elucidating the intricate interplay between host resistance and pathogen virulence strategies.

## Methods and materials

### Understand the molecular code of RMG8 and AVR-Rmg8

The mRNA transcripts encoding the wheat blast-resistant gene Rmg8 were submitted to NCBI in 2023 by Kobe University, Japan^8^. To understand the molecular code of RMG8 and AVR-Rmg8 protein sequences corresponding to these mRNA transcripts, they were retrieved from the GenBank accession (RMG8-V1: BET03927.1, RMG8-V2: BET03928.1) for Rmg8b. Furthermore, different types of AVR-Rmg8 proteins (eI: BBA31636.1, eII: BBE07953.1, eL: BCD40998.1) were sourced from NCBI^30^. The sequences of Rmg8 variants were aligned to identify their similarities and differences. Likewise, the same alignment method was applied to AVR-Rmg8 proteins. BioLuminate 5.3 was employed to align and visualize the protein discrepancies^31^. InterPro was utilized to perform functional analysis of protein sequences by classifying them into families and predicting the presence of domains and critical sites^32^.

### Ancestral proteins of RMG8

To identify proteins closely related to RMG8 proteins, the BLASTp (Basic Local Alignment Search Tool for proteins) was employed^33^. Both variants of RMG8 proteins were subjected to the BLASTp, and proteins were selected based on the conservation of RMG8 proteins. Subsequently, RAxML (Randomized Axelerated Maximum Likelihood) version 8.2.12 was integrated into our study to assess the strength of the relationship between RMG8 and the selected proteins^34^. To enhance the robustness of phylogenetic analysis under maximum likelihood, 1000-time bootstrapping was performed. Operating RAxML on a cluster-based computer with thread-changing capabilities expedited program execution compared to other available phylogenetic analysis tools. Once again, the CDD was utilized to extract the conserved domain information, facilitating a deeper comprehension of the molecular characteristics of RMG8 and its close ancestors^35^. The amino acid composition of RMG8 proteins was analyzed alongside that of closely related proteins using Biopython Version 1.76^36^.

### Pathway and protein-protein interaction analysis

The pathway analysis of Rmg variants was conducted using KEGG^37^. No metabolic pathways information corresponds to *T. aestivum* deposited yet at KEGG. To overcome this problem model plants from monocots grass family *Oryza sativa japonica* (Japanese rice), *Aegilops tauschii* (wheat D) and *Zea mays* (maize) were selected to search metabolic pathways for RMG8 proteins. To run this analysis, the RMG8 sequence of both variants was input as query sequences into the KASS (KEGG Automatic Annotation Server) search system. However, pathways were inferred based on the output tree from RAxML. To investigate the direct or physical interactions of RMG8 with other proteins in *T. aestivum*, protein-protein interaction analysis was performed using computational prediction toolkits such as STRING (Bayesian integration framework)^38^. Since STRING-based predictions may include false positives, particularly in wheat, where curated interaction data are limited, both RMG8 variants were analyzed. To increase reliability, the protein showing the highest sequence homology with RMG8 from the database was incorporated, thereby enhancing the likelihood of true interactions and reducing spurious association.

### Molecular architecture of RMG8 and AVR-Rmg8

RMG8 is a newly revealed blast-resistant gene in *T. aestivum*; no experimental protein structure has been deposited yet. In wet lab experiments, it has been confirmed that RMG8 interacts with AVR-Rmg8, which is an important effector protein of MoT. Importantly, there is no experimental structure available for the AVR-Rmg8 too. Subsequently utilizing Rosetta, the 3D architecture of both RMG8 proteins and AVR-Rmg8 variants was generated^39^. This computational process was executed on a Supermicro built cluster-based computer. Due to the absence of satisfactory homologous structures, an *de novo* structure prediction method was employed based on the AlphaFold provided structures of RMG8 proteins^40^. The use of Rosetta helps us to understand the 50 similar structures of each variant. The quality of final structures were assessed by using UCLA-DOE LAB — SAVES v6.1^41^. The construction of protein structures involved utilizing Rosetta fragment libraries, which integrated experimentally determined structure fragments to guide the exploration of conformational space during the prediction^42,43^.

### The molecular characteristics of RMG8 and AVR-Rmg8

To uncover the biological function of RMG8, analysis was conducted based on the conserved functional domains. Substrate binding clefts in the conserved domains were identified through the screening of the structure and substrate complexes using the binding site module of the Schrodinger package^31^. In the next step, the machine learning algorithms E2EATP and MIB2s were used to know the critical amino acids involved in domain functioning^44,45^. The E2EATP end-to-end deep learning model improved ATP-binding site prediction by extracting informative features from protein sequences. A Metal Ion-Binding Site (MIB) predictor was then used to identify Ca²⁺ binding sites within the calcium-binding domain. The screened MIB-predicted complexes were subsequently subjected to molecular dynamics simulations^46^. Subsequently, the subcellular localization of RMG8 proteins and the presence of transmembrane helices in proteins were studied by Plant-mPLoc and TMHMM − 2.0^47^. It is established that AVR-Rmg8 is an effector protein^48^, but to understand the variation among the AVR-Rmg8, we investigated the effectoromics status of AVR-Rmg8 using EffectorP and SignalP 5.0^49,50^.

### Kinase activity of RMG8

To know the biological activity of RMG8, multiple complexes were prepared for different variants of RMG8 and AVR-Rmg8 proteins. In the beginning, to know the structural discrepancies in AVR-RMG8, structural comparisons were performed with UCSF Chimera. Subsequently, the PDB file of RMG8 was split, and only the PKC domain was used to make complexes. Each complex has a PKC domain, an AVR-Rmg8 variant, and an ATP molecule. After forming the complex virtual screening workflow program, BioLuminate was used to determine the kinase activity of the PKC domain through the initial screening analysis^31,51^. For the final validation of the PKC domain activity, the molecular dynamics simulation was performed using the Desmond package developed by Schrodinger^52,53^.

### Interaction of RMG8 variants and membrane insertion

Previous studies revealed that both variants of RMG8 are required to confirm the resistance against MoT^11,54^. We applied several approaches to know why both variants are required to verify the resistance. The random and selective screenings were performed to capture the interaction between two variants by using the Schrodinger package Bioluminate^31^. A protein-protein docking workflow was used for screening, requiring preprocessing of each protein. To find the best interaction, the program was rotated 70,000 times, selecting the best-refined pose. Membrane insertion of RMG8 complexes was analyzed using the Desmond membrane preparation workflow^46^. The POPC membrane model was picked up to develop the membrane system, and the model was placed based on the domain’s affinities to the membrane component^55^. Molecular dynamics were performed for both complexes in the membrane system at a 200 ns time scale to capture their dynamics in the membrane system.

### Membrane Preparation for eI variant and RMG8 complex

The membrane composition was derived from existing literature on *Arabidopsis thaliana* and nuclear membrane compositions^56^. Three different membrane compositions were tested, each with varying proportions of phosphatidic acid (PA), phosphatidylcholine (PC), phosphatidylglycerol (PG), phosphatidylethanolamine (PE), phosphatidylinositol (PI), sterols, and ceramides^57^. The membrane bilayer was generated using the membrane builder interface within CHARMM-GUI, and a rectangular box system was constructed with TIP3P water interphase^58^. System size determination options were applied to calculate lipid numbers in both leaflets, ensuring membrane homogeneity. The system was built using the replacement method, and Ca²⁺ ions were incorporated via the distance ion-placing method^56^. Finally, the CHARMM36m force field was chosen to obtain input conducive to running in the Desmond package^58^.

### Interaction of RMG8 and eI variant in the membrane system

The RMG8 and AVR-Rmg8 complex and ATP integrated into the membrane. Molecular dynamics simulations with Desmond, using the CHARMM36m force field, studied the complex’s activity within the lipid membrane^58^. During the simulation’s outset, the entire system underwent preprocessing and optimization, addressing bond order, hydrogen replacement, and filling missing side chains^59^. The membrane system was assessed over about 1000 frames, each 1 ps, with periodic boundary conditions. All chemical interactions charged, hydrophobic, electrostatic, van der Waals, and solvent exposure were calculated within a 10 Å orthorhombic box^60,61^. The temperature was maintained at a reference temperature of 300 K, and pressure coefficients of 1 ps were applied to all atoms^62^.

## Supplementary Information

Below is the link to the electronic supplementary material.

Supplementary Material 1

Supplementary Material 2

Supplementary Material 3

Supplementary Material 4

Supplementary Material 5

Supplementary Material 6

Supplementary Material 7

Supplementary Material 8

Supplementary Material 9

Supplementary Material 10

Supplementary Material 11

Supplementary Material 12

## Data Availability

The datasets generated and/or analyzed during the current study are available in the figshare repository at (https:/figshare.com/articles/dataset/Structural_Insights_into_AVR-Rmg8_Recognition_Mechanisms_by_the_Wheat_Blast_Resistance_Gene_i_Rmg8_i_/29558102).
