# Supplementary figures and images for: Structural insights into AVR-Rmg8 recognition mechanisms by the wheat blast resistance gene Rmg8
Source: Sci Rep. 2025 Dec 4;15:45777. doi: 10.1038/s41598-025-28559-5 (PMC12756323; doi:10.1038/s41598-025-28559-5)

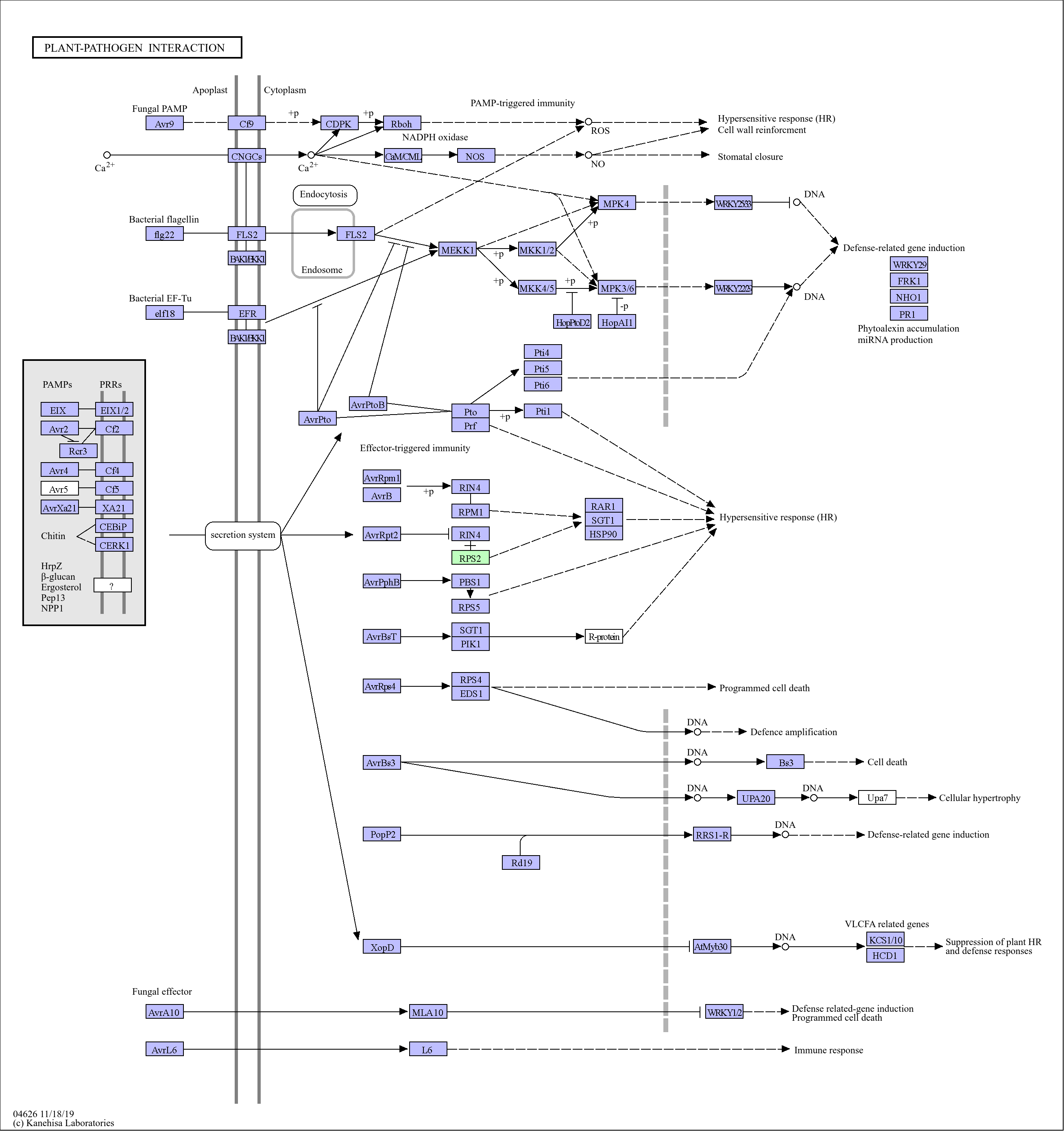

Supplement: Supplementary file 1 — Supplementary Material 1 [file 41598_2025_28559_MOESM1_ESM.png]

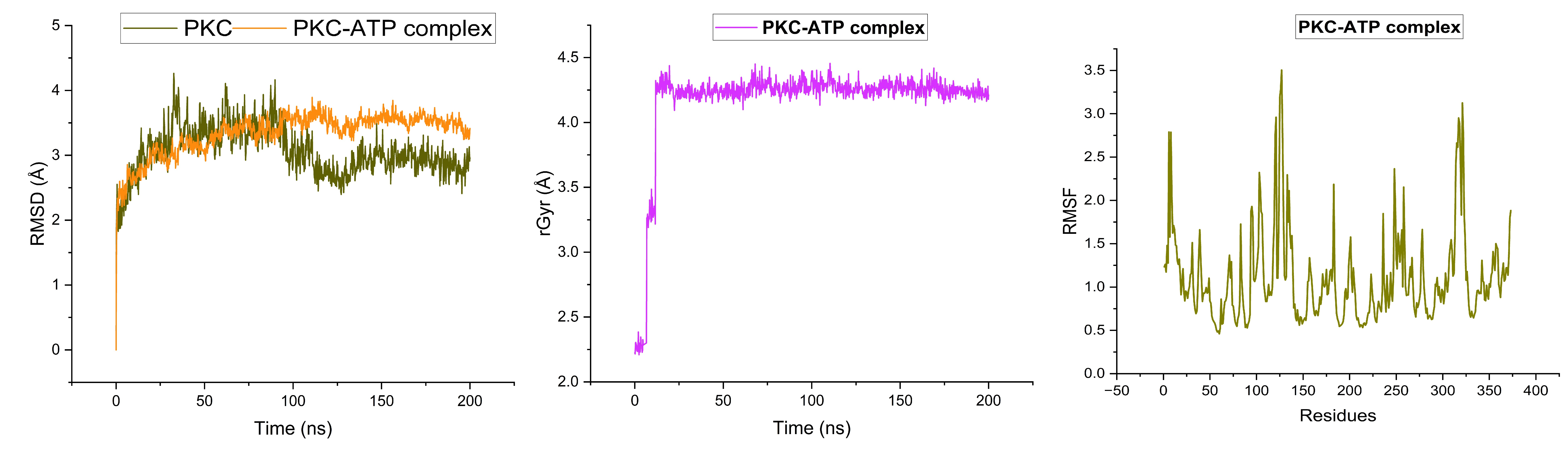

Supplement: Supplementary file 11 — Supplementary Material 11 [file 41598_2025_28559_MOESM11_ESM.png]

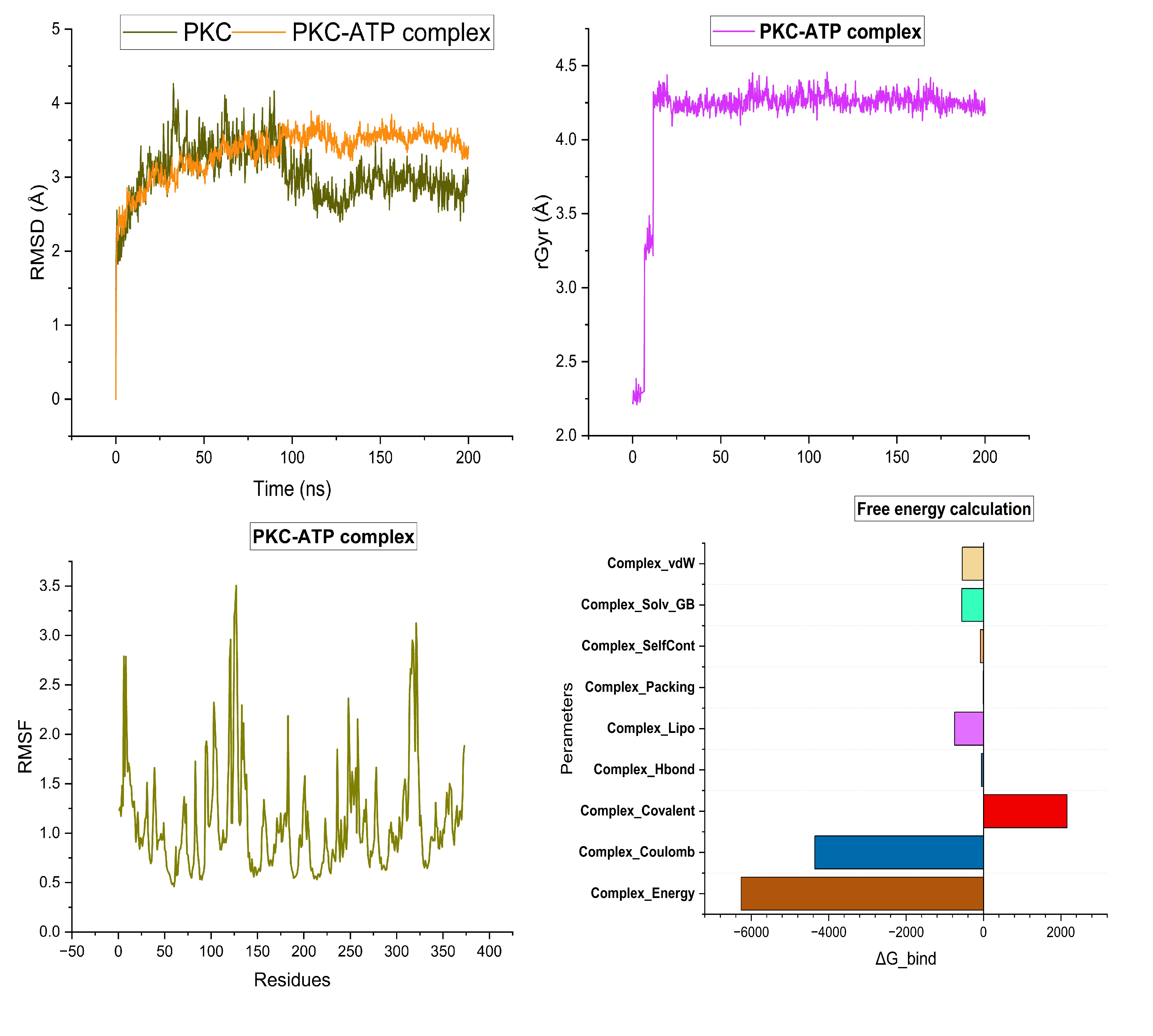

Supplement: Supplementary file 12 — Supplementary Material 12 [file 41598_2025_28559_MOESM12_ESM.png]
