# Supplementary material for: Structural insights into AVR-Rmg8 recognition mechanisms by the wheat blast resistance gene Rmg8
Source: Sci Rep. 2025 Dec 4;15:45777. doi: 10.1038/s41598-025-28559-5 (PMC12756323; doi:10.1038/s41598-025-28559-5)
Supplement: Supplementary file 5 — Supplementary Material 5 [file 41598_2025_28559_MOESM5_ESM.docx]

**Supplemental Materials**

**Fig. S1** Signalling pathway for Rmg8 proteins inferred based on ancestral homology.

**Fig. S2** RMG8 interaction with other signalling proteins.

**Fig. S3** Effectormics status of AVR-Rmg8.

**Fig. S4** RMSD plot that demonstrates the interaction stability between PKC and ATP.

**Table S1** RMSD value for 50 structures of RMG8 V1 variant.

**Table S2** RMSD value for 50 structures of RMG8 V2 variant.

**File S1** 50 structures of RMG8 V1 variant.

**File S2** 50 structures of RMG8 V2 variant.

**File S3** Kinase activity of RMG8 protein.

**File S4** Molecular dynamics of C2 mediated RMG8 variants complex in the membrane system**.**

**File S5** Molecular dynamics of C2-PRT_C mediated RMG8 variants complex in the membrane system.

**File S6.** Membrane dynamics of RMG8 protein while interacting with AVR-Rmg8.

**File S7.** Membrane dynamics data of the RMG8 and Avr-Rmg8 complexes.
